# Multiple site inflammation and acute kidney injury in crush syndrome

**DOI:** 10.3389/fphar.2024.1458997

**Published:** 2024-08-30

**Authors:** Hiroaki Miyauchi, Koshu Okubo, Kiriko Iida, Hiroshi Kawakami, Kentaro Takayama, Yoshio Hayashi, Junji Haruta, Junichi Sasaki, Kaori Hayashi, Junichi Hirahashi

**Affiliations:** ^1^ Department of General Medicine Education, School of Medicine, Keio University, Tokyo, Japan; ^2^ Department of Endocrinology, Metabolism and Nephrology, School of Medicine, Keio University, Tokyo, Japan; ^3^ Division of Food and Nutrition, Graduate School of Human Sciences, Kyoritsu Women’s University, Tokyo, Japan; ^4^ Department of Medicinal Chemistry, Tokyo University of Pharmacy and Life Sciences, Tokyo, Japan; ^5^ Laboratory of Environmental Biochemistry Kyoto Pharmaceutical University, Kyoto, Japan; ^6^ Medical Education Center, School of Medicine, Keio University, Tokyo, Japan; ^7^ Department of Emergency and Critical Care Medicine, School of Medicine, Keio University, Tokyo, Japan

**Keywords:** crush syndrome, rhabdomyolysis, kidney injury, macrophage extracellular trap, muscle damage, intraperitoneal inflammation, systemic inflammatory response syndrome

## Abstract

Crush syndrome, which frequently occurs in earthquake disasters, often leads to rhabdomyolysis induced acute kidney injury (RIAKI). Recent findings indicate that systemic inflammatory response syndrome (SIRS) exacerbates muscle collapse, contributing to RIAKI. The purpose of this study is to investigate the involvement of multiple site inflammation, including intraperitoneal, in crush syndrome. In a mouse model of RIAKI, elevated levels of inflammatory mediators such as TNFα, IL-6, myoglobin, and dsDNA were observed in serum and the peritoneal cavity, peaking earlier in the intraperitoneal cavity than in serum or urine. Our previously developed novel peptide inhibiting leukocyte extracellular traps was administered intraperitoneally and blocked all of these mediators in the intraperitoneal cavity and serum, ameliorating muscle damage and consequent RIAKI. Although further studies are needed to determine whether intraperitoneal inflammation associated with muscle collapse can lead to systemic inflammation, resulting in more severe and prolonged muscle damage and renal injury, early suppression of multiple site inflammation, including intraperitoneal, might be an effective therapeutic target.

## Introduction

Crush syndrome is often fatal because of the acute kidney injury (AKI) secondary to rhabdomyolysis, which results from the physical compression of skeletal muscles. Rhabdomyolysis-induced AKI (RIAKI) is caused by myoglobin and heme released from muscle cells, excess reactive oxygen species (ROS), and cylinder formation in the distal renal tubules. The released myoglobin is filtered by the glomeruli and reaches the proximal tubules. In the proximal tubule, heme is degraded by heme oxygenase-1, and the free iron reacts with hydrogen peroxide resulting in the production of ROS (Zager, 1996). In the distal tubules, myoglobin binds to the Tamm–Horsfall protein, forming a tubular cylinder, which results in tubular obstruction (Petejova and Martinek, 2014).

In addition to direct myoglobin toxicity, the production of inflammatory cytokines and cytotoxic molecules mediated by immune cells has been implicated in enhancing cellular damage and RIAKI. Therefore, it has attracted attention as a therapeutic target. Recent studies have highlighted macrophages as key players in this process. In RIAKI, myoglobin-induced macrophage migration to the renal interstitium and their subsequent activation into a proinflammatory phenotype have been demonstrated. The depletion of macrophages has been reported to decrease renal fibrosis and improve renal protection and survival (Belliere et al., 2015). Moreover, macrophages in the kidneys of patients with RIAKI are sensitive to complement, overexpressing C5aR and CD11b. Renal injury was suppressed in C3 knockout mice, indicating that this ligand-mediated activation of macrophages by the complement system may play a role in renal injury (Boudhabhay et al., 2021).

In 2018, we reported the involvement of macrophage extracellular traps (METs) as a novel mechanism of RIAKI (Okubo et al., 2018). Extracellular traps (ETs), such as neutrophil extracellular traps (NETs) and METs, contribute to host immune defense. However, their dysregulation contributes to autoimmune diseases (Kessenbrock et al., 2009), thrombosis (Brühl et al., 2012), inflammatory diseases (Clark et al., 2007), and severe diseases in patients with COVID-19 (Middleton et al., 2020). Recent studies have demonstrated that systemic inflammatory response syndrome (SIRS), which can occur following muscle injury, can create a vicious cycle that exacerbates muscle damage. This systemic inflammation can eventually lead to renal injury associated with RIAKI and damage to other distant organs (Tu and Li, 2023). Given that intraperitoneal ETs have been implicated in systemic inflammation and distant organ damage (Li et al., 2022), we investigated the existence and association of intraperitoneal ETs and inflammation in crush syndrome.

### Muscle injury induces systemic inflammation, prolonging muscle injury and kidney damage

SIRS often develops in patients with muscle injury, contributing to shock and multiple organ failure (Lenz et al., 2007). Inflammation resulting from infection induces an innate immune response that recognizes infectious pathogens as “non-self,” resulting in sepsis. Conversely, inflammation resulting from muscle injury is sterile inflammation. Sterile inflammation occurs when the immune system senses danger molecules released from damaged or stressed tissues (Zhang and Mosser, 2008). Typically, danger molecules are sequestered intracellularly; however, when tissues are damaged, damage-associated molecular patterns (DAMPs) are released, triggering an immune response by activating pattern recognition receptors (PRPs) on immune cells, leading to systemic inflammation and secondary organ failure (Eppensteiner et al., 2018). Sterile inflammation activates lymphocytes, macrophages, and neutrophils, which enhance local tissue injury by releasing inflammatory cytokines and generating ROS (Queme et al., 2017). Some inflammatory cytokines are also released into the blood and circulate to remote tissues, causing local inflammation in distant organs, which in turn further releases DAMPs, resulting in a vicious cycle of inflammation in remote tissues (Eppensteiner et al., 2018). Although various mechanisms of renal injury have been reported, severe acute inflammatory myositis without trauma has also been reported to induce AKI. Inflammatory myositis causes increased myoglobin levels in blood and urine, suggesting that muscle inflammation contributes to RIAKI (Bosch et al., 2009; Hamel et al., 2015).

**FIGURE 1:**
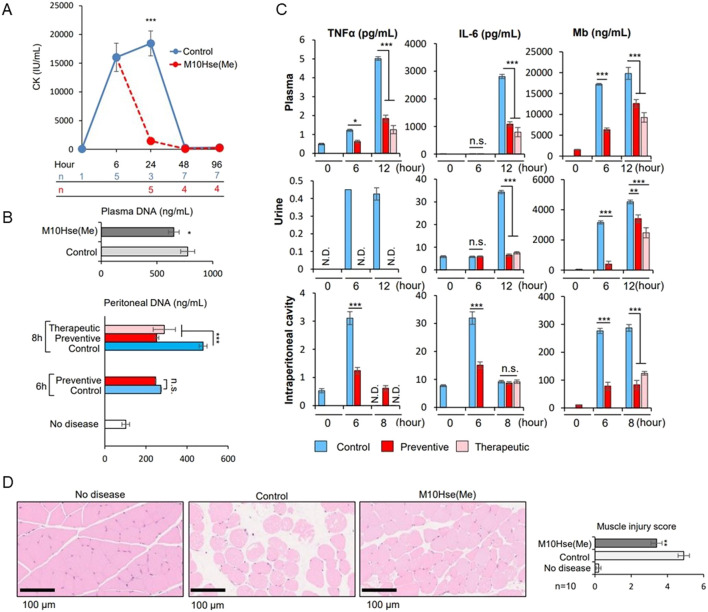
Effect of M10Hse(Me) on muscle protection in rhabdomyolysis and its influence on extracellular traps and inflammatory cytokines, including intraperitoneal inflammation. **(A)** CK levels are markedly elevated by 6 h after muscle injury by injection of 50% glycerol (75 μL) into the bilateral thigh muscles of mice. Intraperitoneal administration of M10Hse(Me) (720 μg) suppresses the increase in CK levels and muscle injury after 24 h. **(B)** Intraperitoneal DNA levels are elevated 6 h after induction of rhabdomyolysis, suggesting intraperitoneal inflammation induced by extracellular traps in the peritoneal cavity; intraperitoneal administration of M10Hse(Me) suppresses the intraperitoneal DNA elevation. **(C)** Inflammatory cytokines such as TNF-α and IL-6 are elevated in the intraperitoneal cavity, plasma, and urine following muscle injury. Elevated myoglobin levels were also observed in the peritoneal cavity. Inflammatory cytokine and myoglobin levels were elevated in the peritoneal cavity before their increase in plasma and urine, suggesting that intraperitoneal inflammation may be involved in the preliminary stage of systemic inflammation and renal injury. **(D)** Intraperitoneal administration of M10Hse(Me) is myoprotective in muscle pathology at 24 h. Intraperitoneal administration of M10Hse(Me) suppressed these factors, acting on early intraperitoneal inflammation and preventing persistent muscle injury and organ damage.

We demonstrated the importance of MET release as a novel mediator of crush syndrome using a glycerol-induced rhabdomyolysis model (Okubo et al., 2018). Using this mouse model, we further examined the mechanism underlying inflammation-induced muscle injury and RIAKI. The injection of glycerol into both thighs of mice induced muscle collapse, which produced a marked increase in CK levels by 6 h which continued to increase until at least 24 h (Fig. 1A). Regarding RIAKI, we previously reported that Cr and BUN levels increased 6 h after glycerol injection, peaked at 24 h, and persisted for more than 96 h (Okubo et al., 2023). In the present study, we detected an increase in dsDNA levels not only in the plasma but also in the intraperitoneal cavity by six or 8 h after glycerol injection, suggesting the formation of intraperitoneal ETs (Fig. 1B). Additionally, we detected inflammatory mediators such as TNFα and IL-6, which are known to be related to autoimmune diseases and acute inflammatory reactions, including COVID-19. Notably, the intraperitoneal concentrations of TNFα and IL-6 peaked at 6 h, whereas their concentrations in plasma and urine peaked at 12 h post-induction (Fig. 1C). Other stress signals tested, including IL-1β, IL-4, IL-12 p70, IL-13, CCL2/MCP-1, and IIFN-γ, were not elevated after glycerol injection (data not shown). These data suggest that intraperitoneal ET formation precedes systemic inflammation. Plasma and urinary myoglobin levels were elevated at 6 h and continued to increase until 12 h (Fig. 1C), suggesting their involvement in RIAKI. Based on previous reports showing that intraperitoneal ETs induced by OVA and adjuvants can lead to systemic inflammation (Li et al., 2022), we attempted to demonstrate that rhabdomyolysis induces the formation of intraperitoneal ETs that mediate systemic inflammation and enhance inflammation in distant organs, resulting in functional impairment.

In a previous study (Okubo et al., 2023), we developed a potent peptide that prevented and ameliorated crush syndrome. We proved in 2016 that the biological multifunctional substance lactoferrin strongly inhibits leukocyte release of extracellular traps, and the mechanism is attributed to the positive charge of lactoferrin that causes chromatin to aggregate (Okubo et al., 2016). Based on these findings, the following structure-activity relationship studies were conducted to develop novel anti-inflammatory therapeutic peptides with leukocyte extracellular trap inhibitory properties. First, we identified a peptide sequence (FK-12) of 12 amino acids rich in positive charge derived from bovine lactoferrin that potently inhibits neutrophil extracellular trap (NET) release *in vitro* using human peripheral polymorphonuclear neutrophils. Further structural modifications based on the alanine scanning mutagenesis method were used to generate multiple peptides with potent NETs inhibitory activity. These multiple peptides were then submitted to the rhabdomyolysis-induced AKI mouse model, and the reno-protective effects of these analogs were evaluated *in vivo*. The results showed that only the candidate drug [M10Hse(Me)], in which the sulfur of Met10 in FK-12 was replaced with oxygen, exhibited superior reno-protective activity and dramatically reduced lethality in the RIAKI mouse model.

In this model, preventive intraperitoneal administration of M10Hse(Me) before glycerol injection did not affect CK levels at 6 h, suggesting that the peptide did not modify the initial induction of the disease (glycerol-induced muscle destruction) (Okubo et al., 2023). Here, we found that the therapeutic administration of M10Hse(Me) 6 h after glycerol injection significantly suppressed CK elevation at 24 h, in contrast to the non-treated group (Figure 1A). Therapeutic administration did not affect Cr and BUN levels at 8 h, but significantly suppressed their increase at 24, 48, and 96 h (Okubo et al., 2023). In the present study, plasma myoglobin concentration after 12 h of glycerol injection was also suppressed by M10Hse(Me) administration (Figure 1C), and muscle pathology analysis at 24 h showed a muscle-protective effect (Figure 1D). These results suggest that the intensity of sustained MET-mediated inflammation from 6 to 24 h determines the fate of further muscle injury and RIAKI. Furthermore, the therapeutic and preventive intraperitoneal administration of M10Hse(Me) suppressed the increase in intraperitoneal DNA levels 8 h after glycerol injection (Figure 1B). The administration of M10Hse(Me) also suppressed TNFα and IL-6 concentrations in plasma and urine, which peaked at 12 h following intraperitoneal injection (Figure 1C). These data suggest that the intraperitoneal administration of M10Hse(Me) suppresses the initial inflammatory response, including intraperitoneal ETs and cytokine bursts, which cause sustained muscle destruction without affecting the initial muscle injury caused by glycerol. In summary, M10Hse(Me) suppresses persistent muscle injury by suppressing subsequent MET-mediated inflammation without affecting the direct muscle injury caused by glycerol, preventing eventual RIAKI and mortality. These findings suggest that the vicious cycle accelerated by muscle injury and systemic inflammation is mediated by intraperitoneal inflammation, which, in turn, prolongs muscle injury and persists in AKI.

### Involvement of peritoneal inflammation in systemic inflammation and organ damage

**FIGURE 2:**
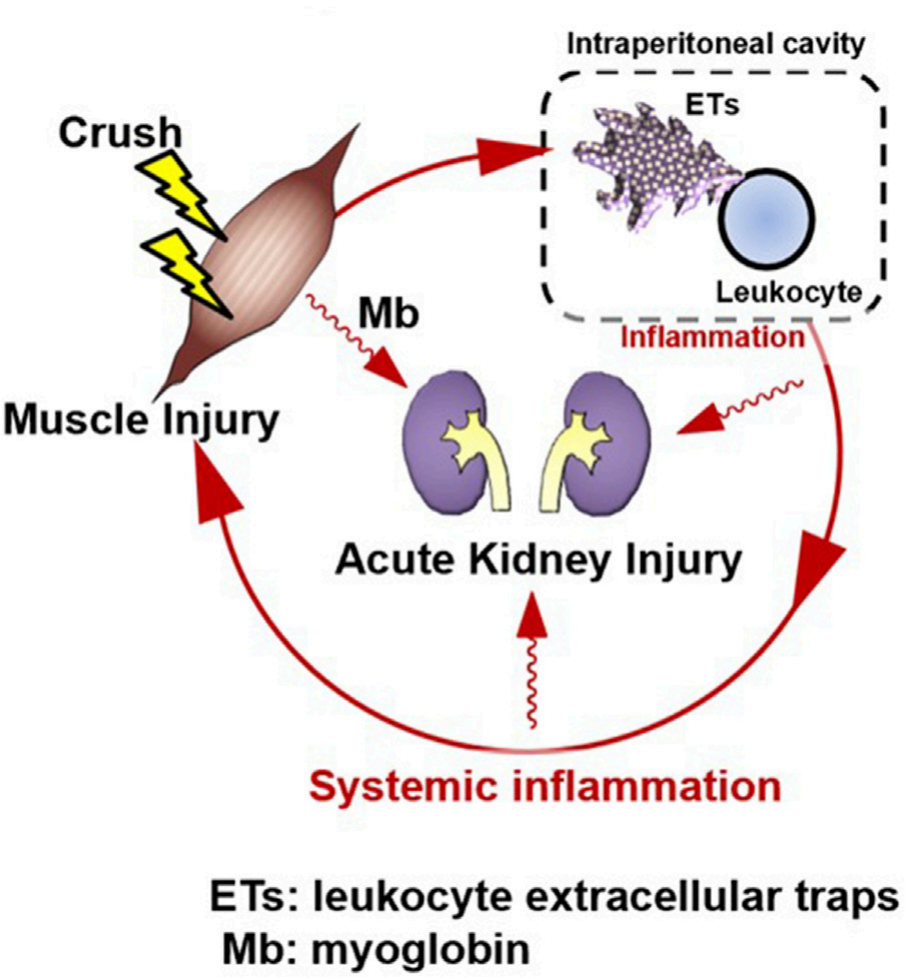
Rhabdomyolysis forms an amplification loop between muscle injury and systemic inflammation via intraperitoneal ETs, resulting in organ damage. Skeletal muscle injury is followed by systemic inflammation; however, intraperitoneal inflammation precedes it. A vicious cycle may form, in which muscle injury induces intraperitoneal inflammation, which then induces systemic inflammation. This induces further muscle injury. Suppression of ETs by M10Hse(Me), which we previously reported, can block the amplification loop of systemic inflammation and muscle damage by suppressing multiple site inflammation. Renal injury, a typical type of organ damage caused by rhabdomyolysis, was also reduced by M10Hse(Me) administration. This suggests that the vicious cycle of systemic inflammation enhances organ damage. Intraperitoneal ETs may be an important therapeutic target to break this vicious cycle.

Intraperitoneal inflammation has been reported to induce systemic inflammation and organ damage during abdominal sepsis (Luo et al., 2014), intraperitoneal tumors (Kanamaru et al., 2018), pancreatitis (Toyokawa et al., 1993), and abdominal surgery (Kanamaru et al., 2018). In localized intraperitoneal inflammation in a porcine model of peritonitis, the level of inflammatory mediators in the peritoneal fluid increased, and inflammatory mediators disseminated into the bloodstream, leading to systemic inflammation (Emr et al., 2014). Intraperitoneal inflammation also causes lung dysfunction in distant organs, which, in turn, produces DAMPs at the site of injury, forming a loop that perpetuates the production of additional inflammatory mediators. In that study, the authors found that reducing intraperitoneal inflammatory cytokines by removing ascites reduced lung injury without affecting endotoxin levels or bacteremia. This suggests that inflammatory ascites may perpetuate organ damage rather than bacterial migration to each organ, leading to organ failure. Furthermore, in the same model, tubular atrophy and leukocyte infiltration also occurred in the kidney, and removal of the ascitic fluid resulted in a significant improvement in histopathological findings (Kubiak et al., 2010). Similar to our data, this report suggests that intraperitoneal inflammation may lead to renal injury and that suppressing intraperitoneal inflammation may prevent organ damage. In a CLP model, NETs were shown to induce systemic inflammation in abdominal sepsis (Luo et al., 2014). In pancreatitis (Toyokawa et al., 1993), intraperitoneal macrophages are activated and may be involved in organ damage. After abdominal surgery (Kanamaru et al., 2018), neutrophils appear in the abdominal cavity and form NETs, which are reported to be involved in peritoneal metastasis. In summary, intraperitoneal inflammation has been suggested to play an important role in sterile inflammation, and ETs may be involved in this phenomenon. In this study we detected the existence of multiple site inflammation including intraperitoneal ETs and cytokine production. Furthermore, the therapeutic effect of M10Hse(Me) on these inflammatory mediators and muscle destruction markers were determined. Our perspective on the pathogenesis of RIAKI assumes that muscle injury is followed by intraperitoneal ET production and inflammation, which in turn precedes systemic inflammation and further amplifies and prolongs muscle injury, resulting in the establishment of Mb-induced AKI, forming a vicious cycle (Figure 2).

## Discussion

Injury to the skeletal muscle leads to the destruction of the muscle sheath, making the muscle fibers brittle and more susceptible to damage. An inflammatory response can lead to further necrosis and worsen the condition (Duan et al., 2021). This amplification of skeletal muscle disintegration and necrosis results in rhabdomyolysis and the release of myocytes into circulation (Vanholder et al., 2000; Furubeppu et al., 2021). Inflammatory cytokine levels are highly elevated in damaged skeletal muscles (Tu et al., 2020), which increases the inflammatory damage. This localized muscle damage subsequently results in systemic inflammation and distant organ damage (Kyriakides et al., 2000).

Acute kidney injury is one of the most common distant organ damage caused by rhabdomyolysis. First, myoglobin is involved as the mechanism of producing RIAKI. Myoglobin is filtered by the glomerulus and generates ROS in tubular epithelial cells, resulting in tubular injury (Zager, 1996; Petejova et al., 2014). It also obstructs the distal tubule by forming a tubular cylinder. Next, the products released by skeletal muscle injury recruit immune cells to the renal interstitium, where they generate inflammatory cytokines, which are thought to cause cellular damage and form an amplification loop. The role of the immune system in RIAKI is poorly understood but is considered crucial. Currently, the only established therapies for RIAKI are fluid replacement and dialysis. Therapies targeting immune cells and inflammatory cytokines are currently being developed; however, they are still in the developmental stage (Knight et al., 2015; Nowak et al., 2017; Hebert et al., 2022). In this report, we propose a novel mechanism of RIAKI that targets ETs. The initial immune response, which includes a surge in inflammatory mediators that release ETs secondary to muscle collapse, occurs primarily intraperitoneally (earlier than in the blood or urine). To the best of our knowledge, this is the first report indicating significant elevations of intraperitoneal cytokines, extracellular traps (ETs), and myoglobin (Mb) during rhabdomyolysis. Based on these findings, we propose therapeutic targeting of ETs and inflammation in multiple sites including intraperitoneal cavity. However, this study has the following limitations. Our data show that in untreated crush syndrome, there are elevated levels of inflammatory cytokines and dsDNA in the circulating blood and intraperitoneal cavity, and that administration of M10Hse(Me) markedly suppresses both elevations and interrupts this vicious cycle between inflammation and muscle damage, but we have not examined in detail how this intraperitoneal inflammation and elevated dsDNA relate to systemic inflammation. Although previous papers have shown that macrophages, rather than neutrophils, are the cells that cause AKI and prognosis in this model of crush syndrome (Belliere et al., 2015), this study does not prove that M10Hse(Me) directly suppresses intraperitoneal METs *in vivo* and therefore does not prove that intraperitoneal inflammation regulates crush syndrome. Currently, the inhibitory effect of FK-12, the basic structure of M10Hse(Me), on inflammation in macrophages has already been reported (Iida et al., 2024), but the detailed mechanism of action of M10Hse(Me) is still under investigation. Developing therapies targeting intraperitoneal ETs could potentially treat RIAKI; however, further studies are needed to validate these findings.

## Data Availability

The original contributions presented in the study are included in the article/supplementary material, further inquiries can be directed to the corresponding author.
